# Supplementary material for: Targeting HDACs in Pancreatic Neuroendocrine Tumor Models
Source: Cells. 2021 Jun 6;10(6):1408. doi: 10.3390/cells10061408 (PMC8228033; doi:10.3390/cells10061408)
Supplement: Supplementary file 1 [file cells-10-01408-s001.zip › cells-1235248-SI.pdf]

cells

MDPI

Supplementary Material

Targeting HDACs in Pancreatic Neuroendocrine Tumor Models

Rosa Lynn Schmitz [1,†], Julia Weissbach [1,†], Jan Kleilein [1], Jessica Bell [2], Stefan Hüttelmaier [2], Fabrice Viol [3], Till Clauditz [4], Patricia Grabowski [5], Helmut Laumen [1], Jonas Rosendahl [1], Patrick Michl [1,*], Jörg Schrader [3,‡] and Sebastian Krug [1,‡]

1 Department of Internal Medicine I, Martin Luther University, D-06120 Halle (Saale), Germany; rosa.schmitz@uk-halle.de (R.L.S.); julia.weissbach@uk-halle.de (J.W.); jan.kleilein@uk-halle.de (J.K.); helmut.laumen@uk-halle.de (H.L.); jonas.rosendahl@uk-halle.de (J.R.); sebastian.krug@uk-halle.de (S.K.)
2 Section Molecular Cell Biology, Institute of Molecular Medicine, Charles Tanford Protein Center, Medical Faculty, Martin Luther University Halle-Wittenberg, D-06120 Halle (Saale), Germany; jbell@ccia.org.au (J.B.); stefan.huettelmaier@medizin.uni-halle.de (S.H.)
3 I. Medical Department, University Medical Center Hamburg-Eppendorf, D-20246 Hamburg, Germany; fabrice.viol@studium.uni-hamburg.de (F.V.); jschrader@uke.de (J.S.)
4 Institute of Pathology, University Medical Center Hamburg-Eppendorf, D-20246 Hamburg, Germany; t.clauditz@uke.de
5 Department of Medical Immunology, Charité Berlin, Corporate Member of Freie Universität Berlin, Humboldt-University Berlin and Berlin Institute of Health, D-13353 Berlin, Germany; patricia.grabowski@charite.de
* Correspondence: patrick.michl@uk-halle.de; Tel.: +345-557-2661; Fax: +345-557-2253
† These authors shared first authorship.
‡ These authors shared last authorship.

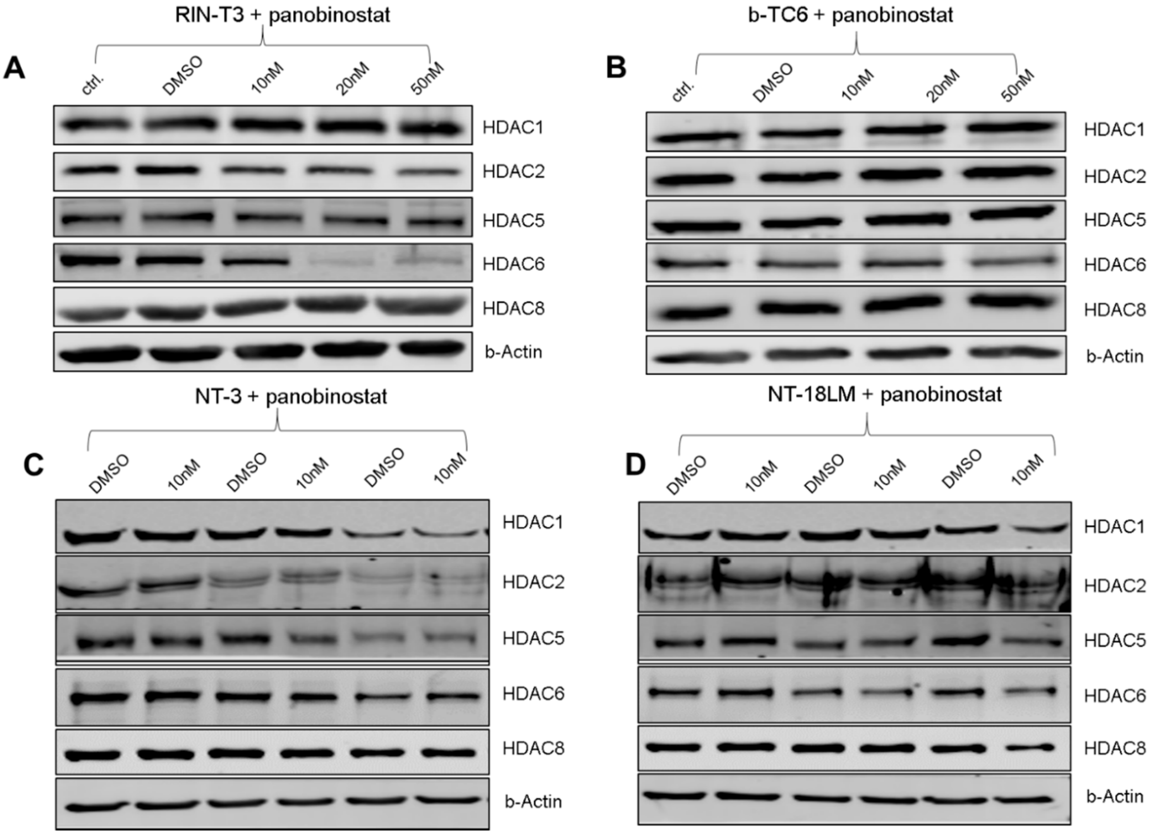

Figure S1. Panobinostat affects endogenous expression of HDACs in murine and human neuroendocrine tumor cells. RIN-T3 (rat) and b-TC6 (mouse) NET cells were treated with increasing concentrations of Panobinostat (PB) (10nM, 20nM, 50nM) and analyzed for HDAC expression. **A**: Western blot analyses of RIN-T3 cells show dose dependent reduction of

endogenous HDAC2 and HDAC6 protein level by PB treatment. **B**: Protein expression of endogenous HDACs were not affected by PB in b-TC6 cells. NT-3 and NT-18LM cells were treated with PB (10nM). Western blot analyses of three independent protein lysates are shown. DMSO served as control treatment. **C**: A reduction of HDAC 5 was observed in the NT-3 cells, whereas no change for HDAC subtypes was present in the NT-18LM cells (**D**).

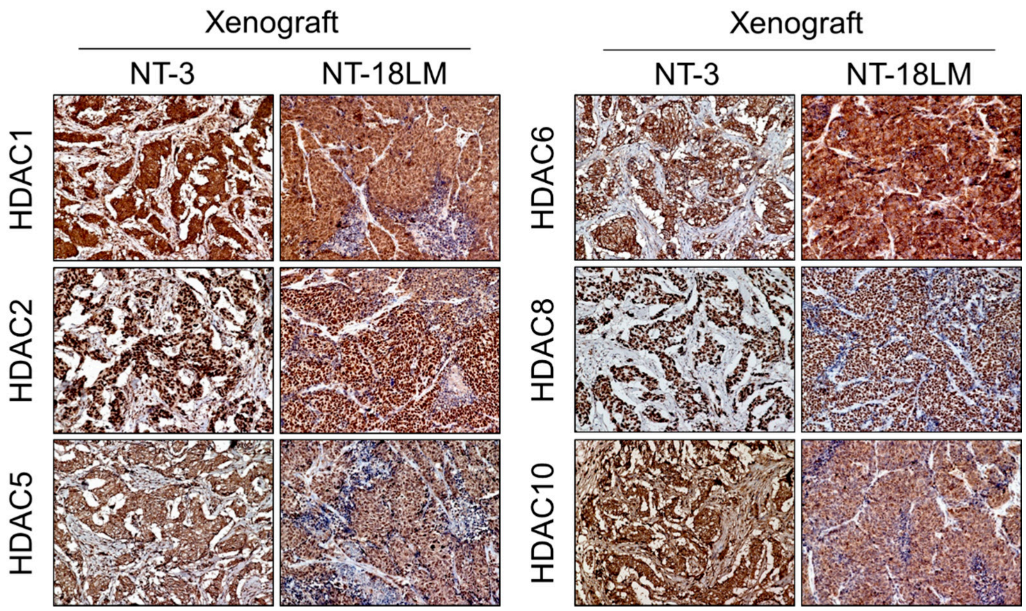

Figure S2. Pattern of HDAC subtypes expression in NT-3 and NT-18LM xenograft tumors. Immunohistochemistry was performed for HDAC1, 2, 5, 6, 8 and 10 in both xenograft tumors. A strong expression for all HDAC subtypes was observed. Representative microscopic images in 40-fold magnification are presented.

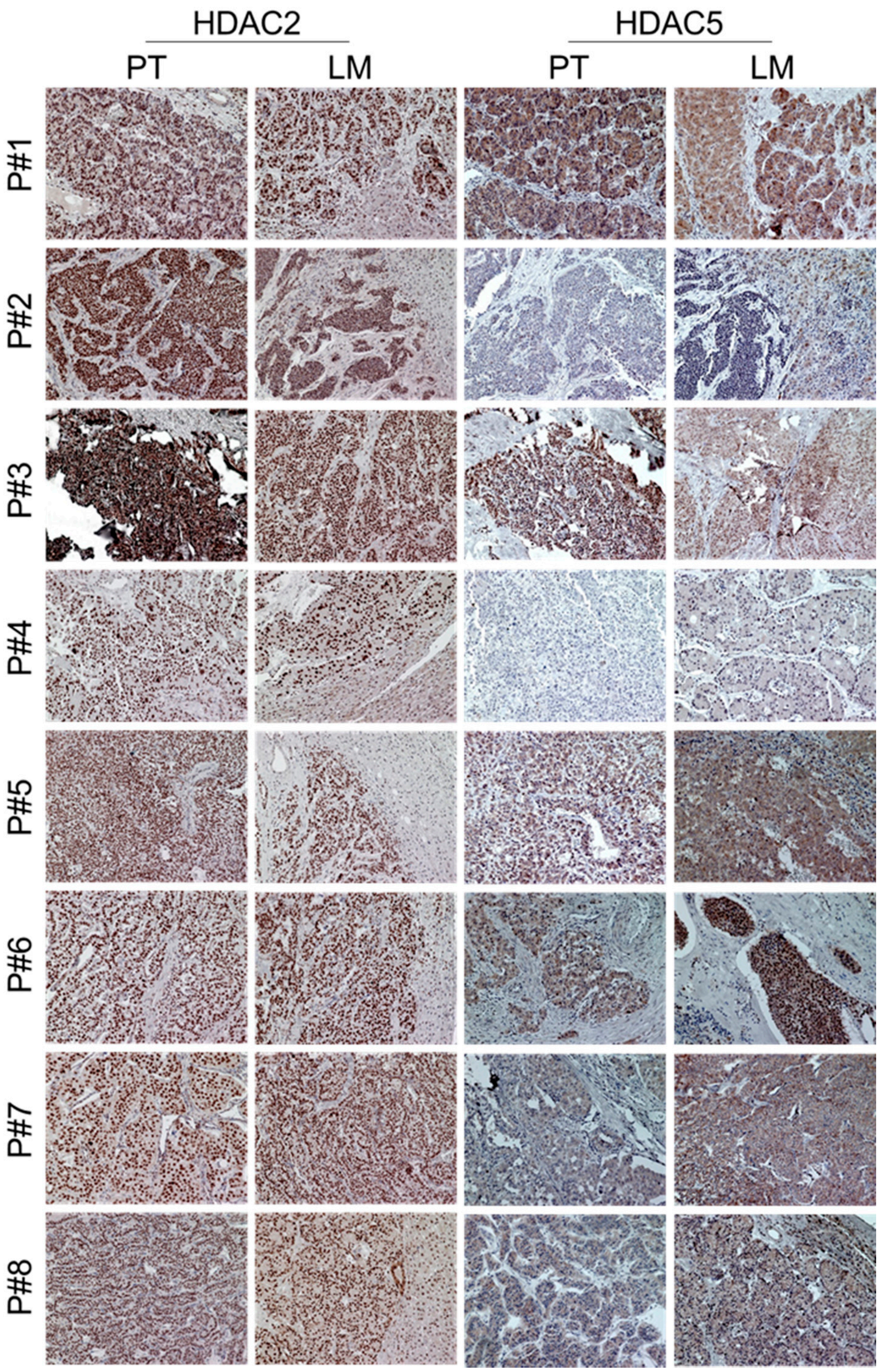

Figure S3. HDAC subtypes expression in human samples of primary tumors and liver metastases. Immunohistochemistry was performed for HDAC2 and HDAC 5 in matched samples of resected pancreatic neuroendocrine primary tumors and liver metastases. Representative microscopic images in 40-fold magnification are presented.

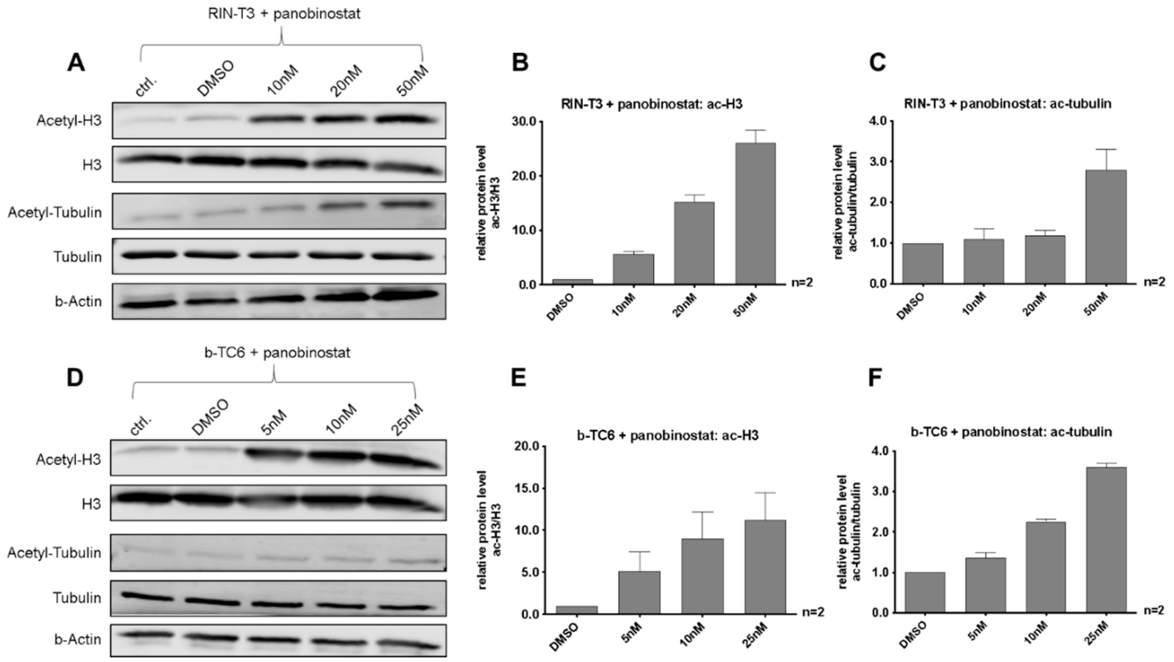

Figure S4. Panobinostat acts as epigenetic modulator. RIN-T3 (rat) and b-TC6 (mouse) NET cells were treated with increasing concentrations of panobinostat (PB) (5nM, 10nM, 20nM, 25nM, 50nM) and analyzed for histone and non histone acetylation. **A**: Western blot analyses of RIN-T3 cells show dose dependent hyperacetylation of histone H3 and non histone tubulin. Western blot quantification revealed dose dependent 5- up to 27-fold upregulation of H3 acetylation (**B**) and 2.8-fold upregulation of tubulin acetylation (**C**) by PB treatment. **D**: Western blot analyses of b-TC6 cells show dose dependent hyperacetylation of histone H3 and non histone tubulin. Western blot quantification revealed dose dependent 5- up to 11-fold upregulation of H3 acetylation (**E**) and 1.2- up to 4.6-fold upregulation of tubulin acetylation (**F**) by PB treatment. Error Bars: SD; N=2; all data were normalized to DMSO which is set to 1; * $p < 0.05$, ** $p < 0.01$, *** $p < 0.001$, according to an unpaired one sample student's t-test.

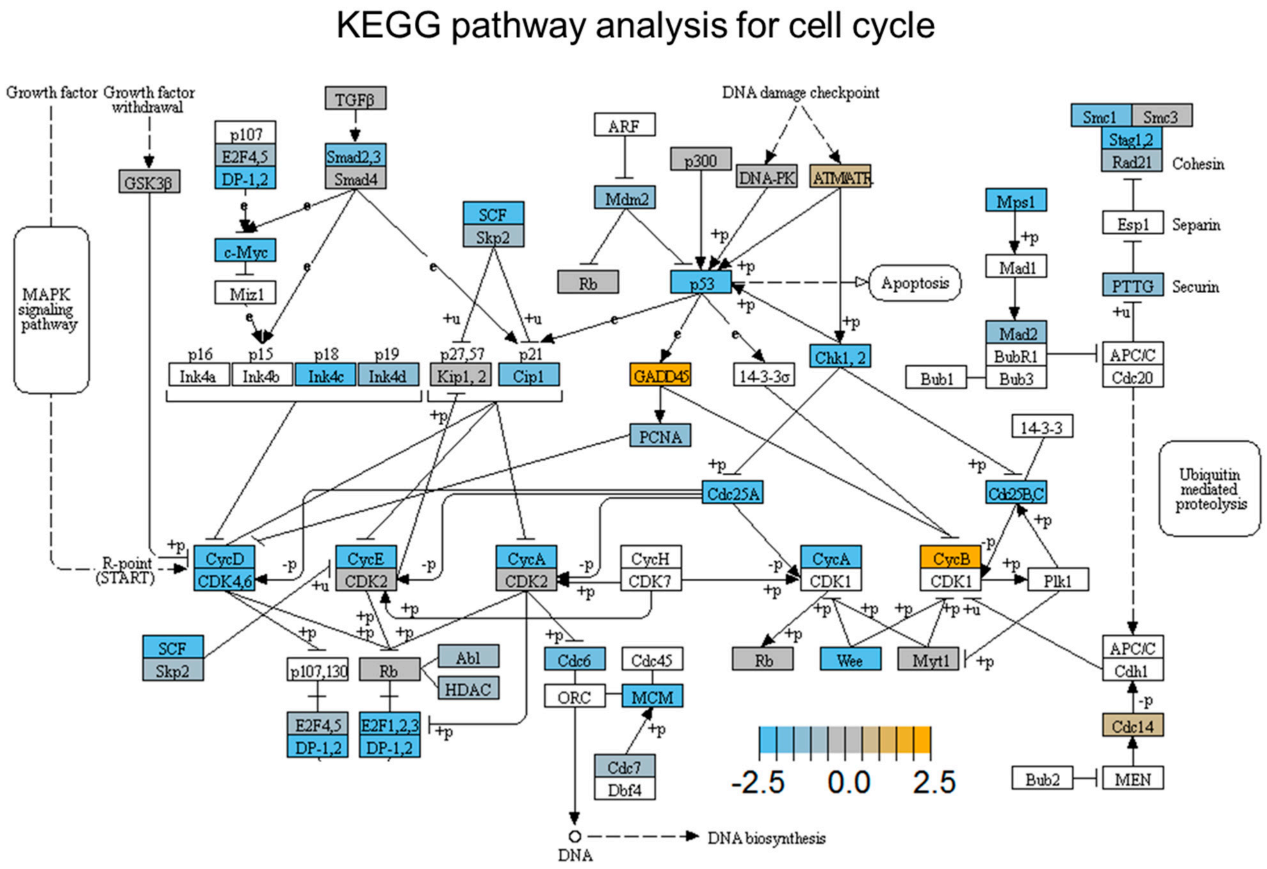

Figure S5. KEGG pathway analysis for cell cycle. Data on KEGG graph rendered by path view verified a strong modulation of genes involved in cell-cycle processes.

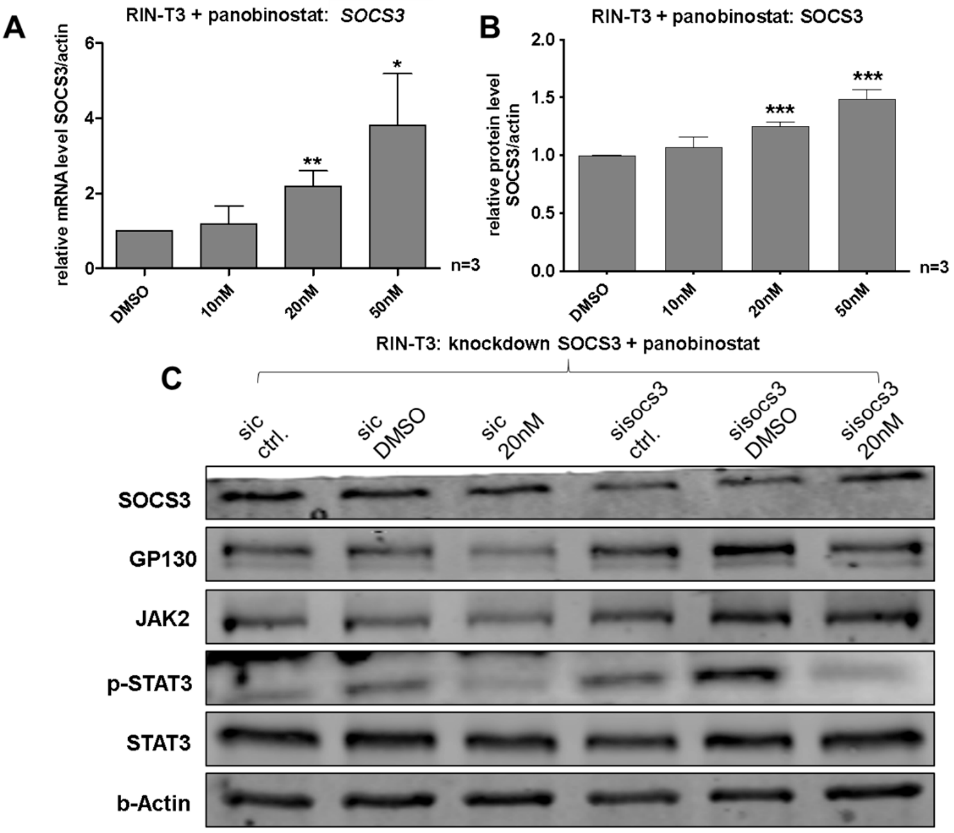

Figure S6. Panobinostat affects the JAK2/STAT3 signaling pathway through SOCS3. **A**+**B**: Quantification of three independent biological replicates shows dose dependent increase of Socs3 on RNA and protein expression level by PB. Western blot analyses of siRNA mediated Socs3 knockdown under PB treatment revealed a slight reduction of gp130 and JAK2 expression and a strong reduction of p-STAT3. Error Bars: SD; $n = 3$; all data were normalized to DMSO which is set to 1; * $p < 0.05$, ** $p < 0.01$, *** $p < 0.001$, according to an unpaired one sample student's t-test.

Supplemental Material S1–S4: Raw data of the gene panels performed by RNA profiling for NT-3 and NT-18LM cells.
